# Shape perception enhances perceived contrast: evidence for excitatory predictive feedback?

**DOI:** 10.1038/srep22944

**Published:** 2016-03-14

**Authors:** Biao Han, Rufin VanRullen

**Affiliations:** 1 Centre National de la Recherche Scientifique, Unité Mixte de Recherche 5549, Faculté de Médecine de Purpan, Toulouse Cedex, 31052, France; 2 Université de Toulouse, Centre de Recherche Cerveau et Cognition, Université Paul Sabatier, Toulouse, 31062, France

## Abstract

Predictive coding theory suggests that predictable responses are “explained away” (i.e., reduced) by feedback. Experimental evidence for feedback inhibition, however, is inconsistent: most neuroimaging studies show reduced activity by predictive feedback, while neurophysiology indicates that most inter-areal cortical feedback is excitatory and targets excitatory neurons. In this study, we asked subjects to judge the luminance of two gray disks containing stimulus outlines: one enabling predictive feedback (a 3D-shape) and one impeding it (random-lines). These outlines were comparable to those used in past neuroimaging studies. All 14 subjects consistently perceived the disk with a 3D-shape stimulus brighter; thus, predictive feedback enhanced perceived contrast. Since early visual cortex activity at the population level has been shown to have a monotonic relationship with subjective contrast perception, we speculate that the perceived contrast enhancement could reflect an increase in neuronal activity. In other words, predictive feedback may have had an excitatory influence on neuronal responses. Control experiments ruled out attention bias, local feature differences and response bias as alternate explanations.

Predictive coding is a form of efficient sensory coding^1^ that relies on the elimination of predictable neuronal responses and thereby the exclusive processing and transmission of unpredicted portions of the sensory input^2^,^3^,^4^,^5^. As such, predictive coding could have important implications for the dynamics of information flow among the different levels of a sensory hierarchy such as the visual cortex.

Standard neuronal implementations of predictive coding assume that the feedback connections carry predictions of expected neural activity and the feedforward connections carry the residual activity between the predictions and initial lower area activity. To carry the residual, the feedforward connections are supposed to be excitatory, whereas to produce the residual the feedback connections are supposed to be inhibitory^3^,^4^. To simplify, standard neuronal models of predictive coding hold that the different hierarchical levels interact by excitatory feedforward carrying residual activity and inhibitory feedback carrying predictions. Recent implementations of predictive coding have divided neurons in each cortical area into two sub-populations, one coding for predictions/representations and one for prediction errors^4^,^6^. These models suggested that only error units would be suppressed through either direct or indirect inhibition from the prediction units; the prediction/representation units, on the other hand, may actually be enhanced by predictive feedback^6^,^7^. Since theory must follow fact, it appears important to investigate the overall perceptual effect of feedback in predictive coding: is it excitatory or inhibitory? Neurophysiology and neuroimaging provide converging supporting evidence for the hierarchical structure and excitatory feedforward connections of predictive coding models^8^,^9^,^10^,^11^, but the experimental data are less unanimous regarding the inhibitory or excitatory nature of predictive feedback^12^: most neuroimaging studies show reduced activity by predictive feedback^13^,^14^,^15^,^16^, while neurophysiology indicates that most inter-areal cortical feedback is excitatory and targets mostly on the lower area excitatory neurons^17^,^18^,^19^,^20^,^21^,^22^. In summary, the experimental literature does not clearly and unambiguously support the notion of inhibitory feedback, which is nonetheless an integral part of many models of predictive coding.

Here, we employed a psychophysical approach to investigate the properties of predictive coding. To produce predictive feedback, we employed similar stimuli as in Murray *et al*.: 3D-shape outlines and random-lines versions of the same stimuli^13^. The former can be easily recognized, and should thus normally produce more predictive feedback than the latter. The two kinds of stimuli (black 3D shape and black random lines) were displayed on gray disks simultaneously on the left and right of a fixation point on a black background. Subjects were asked to compare the luminance of the two disks (report the side of the brightest disk). The 3D-shape disk was perceived systematically brighter than the random-lines disk (i.e., its contrast relative to the black lines and screen background was higher than the contrast of the random-lines disk). Since there is experimental evidence suggesting a monotonic relationship between perceived contrast and neuronal activity in early visual areas^23^,^24^, we speculate that, at least at the moment at which subjects made their perceptual decision about local contrast, predictive feedback was excitatory rather than inhibitory.

## Results

### Main Experiment: luminance judgment

**Figure 1:**
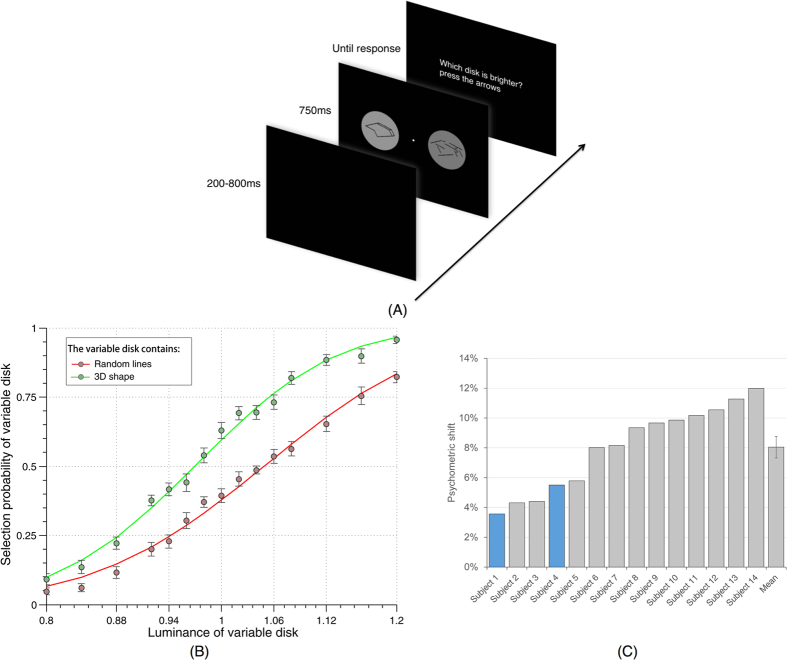
Main experiment and results. (**A**) Experimental paradigm. Each trial consisted of a 200–800ms blank screen, a 750ms stimulus screen and a response screen that remained visible until the response was provided. The stimulus screen consisted of a fixation point, one circular gray disk with a 3D-shape stimulus and another with a random-lines stimulus (with randomized positions on the left and right of fixation for every trial). One disk had a fixed contrast level and the other a variable contrast value around that level (randomly assigned on every trial). Subjects were instructed to compare the luminance of the two disks. No feedback was given after the response. (**B**) Comparison of the grand average psychometric functions (when pooling data over all subjects). Each curve represents the selection probability of the variable disk when this disk contained the 3D-shape (green) or the random-lines stimulus (red). Error bars represent standard error of the mean (SEM) across subjects (**C**) Psychometric shift for each subject and mean across subjects. Psychometric shift was defined as the difference between the two psychometric functions at 50% selection probability. All 14 subjects showed a positive effect, with the disk behind the 3D-shape stimulus perceived brighter against the black background than the one behind the random-lines. Subjects 1 and 4, marked by colored bars, performed the experiment while their eye position was monitored, and any eye movement or break of fixation discarded. Error bar represents SEM.

Participants (N = 14) were instructed to fixate on the fixation point and judge the luminance of two gray disks on a black background on the left or right of fixation; each disk had either a black 3D-shape or a black random-lines pattern (randomly assigned) superimposed in its center (Fig. 1A). As these stimuli differentially activate higher visual areas (such as the lateral occipital complex, LOC^13^), one can reasonably expect different amounts of predictive feedback for the two locations^13^, with more feedback towards the 3D-shape disk. Since anatomical evidence shows that feedback connections are strongly divergent^25^, we reasoned that the influence of predictive feedback might be measurable over the entire disk. Excitatory feedback would increase neural responses to both the disk and stimulus outline, making the gray disk appear brighter and the black outline appear relatively darker (resulting in an increase of total contrast), while inhibitory feedback would decrease both neural responses, making the disk darker and the outline brighter (and reducing total contrast). We reasoned, however, that the brightness over the large area of the gray disks may be easier to judge than the darkness of the narrow lines and contours, and thus we asked the participants to report the side of the disk that they perceived as brighter (after the stimuli offset, they received the instruction “which disk was brighter?”, and responded via button press).

In each block of trials, one disk type was assigned with a fixed luminance value, while the other disk was assigned with a variable value around that level, different on each trial. The positions of the fixed-luminance and variable-luminance disks (and thus of the 3D-shape and random-lines stimuli) were randomly assigned in each trial. Two psychometric functions were computed from the data, one for blocks in which the random-lines disks had variable luminance values, and one for the other block type in which the 3D-shape disks had variable luminance values. We finally compared these two psychometric functions: the psychometric shift was defined as the difference between the two psychometric thresholds (variable luminance value at which selection probability reaches 50%). A positive psychometric shift would suggest that the luminance of the random-lines disk at which it is perceived equiluminant to the fixed-luminance 3D-shape disk is higher than the luminance of the 3D-shape disk at which it is perceived equiluminant to the fixed-luminance random-lines disk. In simpler terms, a positive effect indicates that 3D-shape disks are perceived as brighter than random-lines disks, while a negative effect implies the opposite relation.

Results showed a positive effect for all 14 subjects, i.e. they perceived 3D-shape disks brighter than random-lines disks (Fig. 1B,C). The psychometric shift was 8.04% ± 2.82% (average ± standard deviation across subjects) normalized luminance and the grand average psychometric shift (when pooling data over all subjects) was 7.93%. A student’s paired t test for the psychometric shift shows t(13) = 10.69, p < 8.29 × 10^−8^ with a confidence interval of (6.42%, 9.67%). This effect was unlikely to be due to eye movements or faulty fixation: in two subjects (indicated in Fig. 1C by colored bars) eye position was monitored by an eye-tracker and any trial with sizeable eye movements were discarded; these two subjects still produced positive psychometric shift (3.57% and 5.51%) that were well within the range of the group. Since luminance/contrast discrimination judgments are linked to neuronal activity in early visual cortical areas^23^,^24^, these results indicate that at the moment at which subjects made a decision about luminance/contrast discrimination, the 3D-shape had presumably produced more neuronal activity in early cortical areas than the random-lines stimulus. As the 3D-shape is more recognizable than the random-lines and thus more likely to induce predictive feedback signals, we tentatively conclude that predictive feedback had an excitatory effect on neuronal activity in early visual cortex. However, we also tested several alternative explanations.

### Control experiment: attention bias

**Figure 2:**
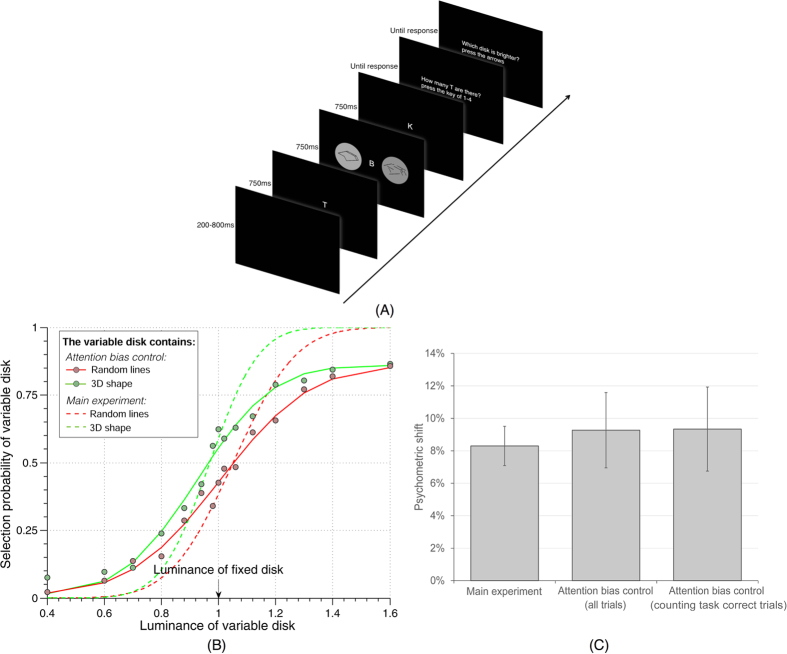
Attention bias control (**A**) Experimental paradigm. Each trial consisted of a 200–800ms blank screen, a 2250ms letter RSVP sequence in the center, a 750ms stimulus screen starting 750ms after the beginning of the letter RSVP, and two successive response screens, each presented until a response was provided. The RSVP sequence displayed randomly drawn letters every 150ms (the same letter could not appear twice in a row). The stimulus screen was the same as in the main experiment, expect for the replacement of the fixation point by the RSVP sequence. Subjects were instructed to first count the number of letters “T” in the RSVP, and (as a secondary task) to compare the luminance of the two disks. Negative auditory feedback was given after every mistake in the counting task. (**B**) Comparison of grand average psychometric functions for the same subjects in the attention bias control (solid lines) and in the main experiment (dashed lines). (**C**) Comparison of psychometric shift for the same subjects in the main experiment and during the attention bias control, either including all trials, or only those in which the counting task was performed correctly. Similar psychometric shift was obtained for all conditions, indicating that attention bias is unlikely to explain our findings. Error bars represent SEM.

An obvious possible confound with our experimental design could be a systematic attention bias towards 3D-shape stimuli. Indeed, previous fMRI studies showed that attention can increase activity in early visual cortical areas^26^,^27^ and alter stimulus appearance including perceived contrast^28^. Is the enhanced perceived contrast for 3D shapes simply a product of increased attention? If this was the case, then one would expect the psychometric shift to decrease when attention is diverted from the peripheral disks using a challenging central task^29^. We thus replaced the fixation point with a rapid serial visual presentation (RSVP) stream of letters. The observers (a subset of participants from the main experiment; N = 7) were instructed to count the number of occurrences (from 1 to 4) of the letter “T” (Fig. 2A), a task known to demand important attentional resources^30^,^31^. To ensure that attention was properly engaged by this central task, we used a presentation speed (6.67 letters/s) that made the task highly challenging (correct rate, 72.48% ± 12.37%, average ± standard deviation across subjects). Participants were instructed to prioritize the counting task and to respond to it first; negative auditory feedback was given after every mistake in this counting task.

Two psychometric functions were generated using the same method as in the main experiment, and compared with the psychometric functions obtained from the same participants during the main experiment (Fig. 2B,C). The psychometric functions had significantly shallower slopes (as measured by the standard deviation of a fitted cumulative normal distribution) than in the main experiment (attention bias control *vs*. main experiment 0.33 ± 0.13 *vs*. 0.16 ± 0.07, average ± standard deviation, t(13) = 4.7, p < 4.23 × 10^−4^, the psychometric function with 3D-shape disk as the variable-luminance disk and the psychometric function with random-lines disk as the variable-luminance disk were analyzed jointly, and 14 pairs of standard deviation values were thus compared for the analysis), suggesting that attention was properly engaged and that subjects were therefore less sensitive to contrast differences^29^. Given that attention was significantly engaged in the central counting task, and regardless of the magnitude of this engagement (i.e., even if only a portion of attentional resources was engaged), an attentional account of our previously observed contrast perception shift should predict that the shift would decrease during the dual-task condition. However, the psychometric shift was not decreased (if anything, it even increased marginally): across subjects, the psychometric shift for this control experiment was 9.27% ± 6.13% (average ± standard deviation across subjects) when including all trials, and 9.34% ± 6.86% when including only those trials in which the counting task was performed correctly (and thus attention was presumably more efficiently engaged); this is to be compared with a psychometric shift of 8.3% ± 3.2% during the main experiment. Paired t-tests showed that the result differences between the control experiment and the main experiment were not significant (including all trials *vs*. main experiment: t(6) = 0.467, p > 0.65; counting task correct trials *vs*. main experiment: t(6) = 0.407, p > 0.69). The grand average psychometric shift (when the psychometric functions were computed from the grand-average data across the seven participants) was 8.82% for all trials, and 8.70% for counting-task correct trials, relative to a psychometric shift of 8.18% during the main experiment.

### Control experiment: local features

We tested yet another alternative interpretation: that low-level local features altered the perceived contrast. Even though the paired 3D-shape and random-lines stimuli have the same number of line segments, comparable line orientations, retinotopic distribution and overall luminance, they also differ in some respects, for example the presence of corners and line junctions in 3D-shapes only. It is conceivable that such local features could influence the processing of local contrast, and that in turn this local alteration of perceived contrast could propagate to the entire disk via filling-in mechanisms. This local contrast alteration mechanism, however, is different from the postulated excitatory feedback effect, since the latter is assumed to depend on the entire shape and thus to be more global in nature. Thus, the two alternative accounts make different predictions about the consequence of changing the contrast polarity of the stimulus outline (black *vs*. white) while keeping the disk luminance (gray) and the screen background luminance (black) unchanged. Indeed, if local features are affecting contrast perception locally, then a white outline on a gray disk (instead of a black outline on a gray disk, as in the main experiment) should result in a reversed contrast effect (3D-shape disk perceived darker than the random-lines disk). On the other hand, the effect of global feedback should not solely depend on the luminance of the stimulus outline (black or white), but also on the contrast between the (gray) disk and its (black) background; if that contrast does not change, the effect of global feedback might be expected to decrease, but should not fully reverse. To distinguish between these alternatives, in this control experiment we replaced the black outline of the 3D-shape and random-lines with white outlines (keeping the disks gray and the screen background black), and asked subjects to perform the same comparison task as in the main experiment (judge which of the two disks is brighter).

**Figure 3:**
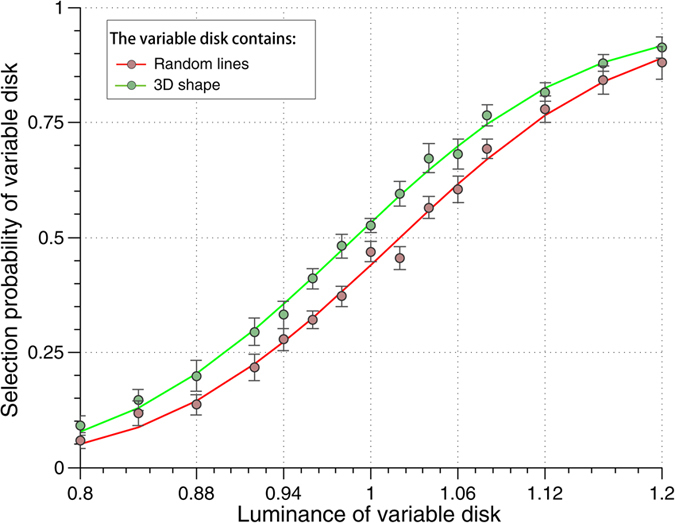
Comparison of the grand average psychometric functions in the “local features” control experiment. In this experiment, the contrast polarity of the stimulus outline was reversed (from black to white) to evaluate the contribution of local features on psychometric shift. While the grand average psychometric shift was reduced, it remained positive (p < 0.05), and did not fully reverse (p < 2.15 × 10^−5^) as would have been predicted if local features were responsible for the entire effect. Error bars represent SEM.

We found that the effect was not reversed by the change of contrast polarity (Fig. 3). The psychometric shift for this control experiment was 3.23% ± 4.44% (average ± standard deviation across subjects; N=10 including 4 participants from the main experiment); the grand average psychometric shift (when the psychometric functions were computed from the grand-average data across participants) was 3.1%. A one-sample Student’s t-test showed that this effect was incompatible with a full reversal (null hypothesis of a psychometric shift of -8.04%, based on the results reported in Fig. 1; t(9) = 8.03 , p < 2.15 × 10^−5^); in fact, this effect was still greater than zero (p < 0.05). This implies that the local contrast polarity is not the sole determinant of the observed effect, and that global feedback must also contribute to it. Therefore, our interpretation of an excitatory feedback still remains viable.

### Control experiment: response bias

**Figure 4:**
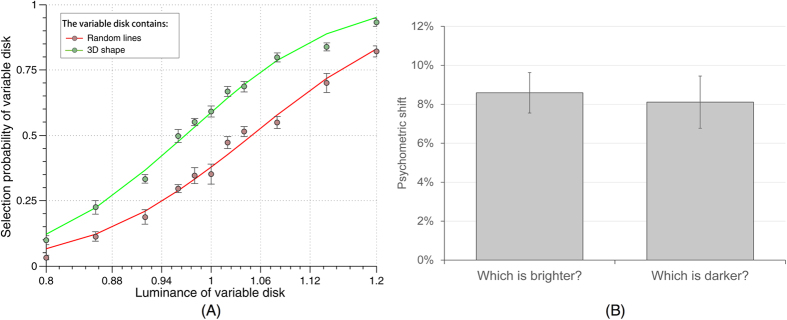
(**A**) Comparison of the grand average psychometric functions in the “response bias” control. (**B**) Comparison of mean psychometric shift for the same subjects in the main experiment and the “response bias” control. In this experiment, the response instruction was reversed (report the darker disk) to measure the influence of a possible response bias. Psychometric shifts were similar in the two conditions (t-test, p > 0.78), indicating that response bias is unlikely to play any major role in the effect. Error bars represent SEM.

We also tested the possible influence of a response bias. One might imagine that when observers do not truly perceive any contrast difference between the 3D-shape and the random-lines disks, but are still confronted with a forced choice between two responses, they could be inclined to systematically choose the one stimulus that they recognized (i.e. the 3D-shape). If this was the case, however, reversing the task instructions (asking “which disk was darker?” instead of “which disk was brighter?”) should not affect this response bias, and should thus produce a reversed psychometric shift (3D-shape disk perceived *darker* than random-lines disk). We re-tested seven participants from the main experiment using these reversed instructions (Fig. 4). None of them showed a reversed effect. The psychometric shift was 8.11% ± 3.54% (average ± standard deviation across subjects), compared with 8.59% ± 2.75% for the same subjects during the main experiment. A paired t-test showed that the differences were not significant (t(6) = 0.2891, p > 0.78). The grand average psychometric shift was 8.06% compared with 8.46% for the main experiment. Thus, response bias is unlikely to account for our findings.

### Control experiment: same/different judgment

**Figure 5:**
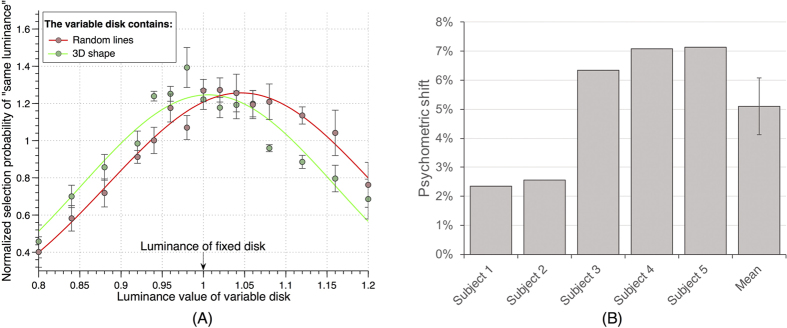
(**A**) Comparison of the grand average psychometric functions in the “same/different” experiment. In this experiment, we instructed subjects to report whether the two disks had the same or different luminance. By comparing the distribution of normalized “same luminance” responses (normalized by mean response probability) on different types of trials (3D-shape or random-lines inside of the variable-luminance disk), we could determine which disk was perceived brighter. The right-shift of the “same” response distribution with random lines inside of the variable disk (or the left-shift of the “same” response distribution with 3D shape inside of the variable disk) indicates that 3D shape enhanced perceived contrast/luminance. (**B**) Psychometric shift for each subject and mean across subjects. Psychometric shift was defined as the difference between the peaks of the two psychometric functions. All 5 subjects showed a positive effect, with a right-shift of the “same” response distribution with random lines inside of the variable disk. Error bar represents SEM across subjects.

Finally, as an even more stringent test against response bias, we instructed subjects (N = 5) to perform a same/different luminance judgment task (asking “Did the two disks have the same luminance?” at the end of each trial). Any response bias towards either the 3D shape or the random lines stimulus would not be expected to affect responses in this sort of task. For different types of trials (3D-shape or random-lines inside of the variable-luminance disk), we measured the probability of “same luminance” response as a function of the luminance of the variable-luminance disk. If shape perception truly has an effect on contrast/luminance perception, we should expect a shift of the distributions. Indeed, we found a right-shift of the distribution of “same” responses when random lines were inside of the variable-luminance disk (relative to the distribution of “same” responses when 3D shape were inside of the variable disk), indicating that 3D shape enhanced perceived contrast/luminance (Fig. 5). By fitting each distribution to a Gaussian function and comparing their peaks, we found an average psychometric shift of 5.10% ± 2.43% (average ± standard deviation across subjects). This psychometric shift corresponded to a p value of 0.0093 with a confidence interval of (2.08%, 8.12%). To compute the grand average psychometric shift, we first normalized the response distributions of each subject relative to their mean value across all possible variable luminance, and then we fitted the average normalized distributions with Gaussian functions. The grand average psychometric shift over 5 subjects was 3.98%. Since this measurement is less prone to response biases, we thus re-confirmed our findings with convergent evidence.

## Discussion

In the present study, consistent behavioral responses of 14 subjects (Fig. 1) revealed that the disk behind the 3D-shape stimulus (which could be easily recognized, and give rise to predictive feedback) was perceived brighter against the black background than the one behind the random-lines (meaningless) stimulus. Given previous evidence suggesting a monotonic relationship between contrast perception and neural activity in early visual areas^23^,^24^, we tentatively interpret these results as evidence that predictive feedback had an excitatory effect on sensory activity, at least at the time point at which contrast perception was established.

We performed four control experiments to rule out alternative explanations of our results. By replacing the center fixation point with an attentionally demanding task (letter RSVP), we obtained similar psychometric shifts for all conditions, indicating that attention bias was unlikely to explain our findings (Fig. 2). In the main experiment, two contrasts could have contributed to the perceived disk luminance: a local one reflecting the luminance difference between stimulus lines and disk, and a more global one caused by the luminance difference between disk and screen background. Both contrasts could have been affected by predictive feedback (e.g., due to divergent feedback connections); but in addition, the local contrast could also have been modulated by more local confounding factors, such as systematic physical differences in the random lines *vs*. 3D-shapes stimuli (although the number of lines and corresponding numbers of pixels were equated, higher-order statistics reflecting inter-pixel relations were not equated). To test if the local factors could solely account for our results, we examined the relative contribution of local and global contrast to the perceived disk luminance by reversing the polarity of the stimuli outline, from black to white. This operation reversed the direction of the contribution from local contrast: if it had previously resulted in the disk being perceived brighter, it should have now caused it to be perceived darker. We showed, however, that psychometric shifts did not fully reverse, indicating that local factors were unlikely to explain all of our findings (Fig. 3). Finally, we used two separate experimental manipulations to assess the effect of response biases on our results: we modified the response instructions (asking “which disk was darker?” instead of “which disk was brighter?”, Fig. 4), and in a separate control we changed the subjects’ task (to a same/different perception task, by asking “Did the two disks have the same luminance?”, Fig. 5). The comparable psychometric shifts obtained regardless of task instructions indicated that response biases were unlikely to explain our findings.

These results concur with neurophysiological evidence that cortico-cortical feedback connections are mainly excitatory^17^,^18^,^19^,^20^,^21^. However, they also appear to contradict neuro-imaging evidence suggesting that predictive feedback is inhibitory, using a similar paradigm and the same set of stimuli as in the present study^13^. The major difference between our study and that of Murray *et al*.^13^ is the dependent variable used to estimate neural activity: perceived contrast *vs*. BOLD activity. The existence of a monotonic relationship between contrast and neural activity in early visual cortical areas has been well established in neurophysiology^23^. The contrast response function of striate cortex neurons has been directly measured in cat and monkey^32^. In human primary visual cortex, contrast is directly related to BOLD responses^33^, and psychophysical contrast judgments (i.e., perceived contrast) are also linked to BOLD responses in visual areas V1, V2d, V3d and V3A^24^. Selective contrast tuning exists for some V4 neurons, however, contrast still has a monotonic and positive relationship with the activity of overall V4 neuron populations^34^. It thus appears reasonable to use perceived contrast as a proxy for overall neuronal activity in early visual cortex. On the other hand, perceived contrast and BOLD activity certainly differ in terms of their temporal resolution: perceptual decisions can be made within a few hundred milliseconds, whereas BOLD signals have a slower time course and a much poorer temporal resolution (on the order of seconds) due to the nature of the hemodynamic response function. Thus, it is possible to envision that predictive feedback could play an excitatory role during early stages of stimulus processing, and yet have a long-lasting inhibitory effect on subsequent neuronal activity.

With the same set of stimuli but complementary methods, the combination of our psychophysical study and previous neuro-imaging results^13^ thus highlights a possibly more comprehensive temporal profile for predictive feedback. But, is this profile universal? Is it comparable across all brain regions? Summerfield *et al*. and Egner *et al*. investigated predictive feedback by measuring BOLD responses in FFA^35^,^36^. With 750ms-long face images, Egner *et al*. showed that FFA responses decreased with high prior expectation compared to low expectation. On the other hand, with masked 100ms-long face images, Summerfield *et al*. found that FFA responses increased during a face-related task compared to a non-face-related task. Even though none of these authors explicitly linked these two studies with respect to stimulus timing, the corresponding time-line of predictive feedback in FFA appears compatible with our hypothesis. At the opposite end of the visual system, Olsen *et al*. showed that the corticothalamic feedback from layer 6 of mouse V1 to lateral geniculate nucleus (LGN) played an inhibitory role: a large proportion of visually evoked activity in LGN relay neurons was inhibited when driving V1 layer 6 neurons optogenetically^37^. Nonetheless, anatomical evidence suggests that direct feedback connections from visual cortex to LGN relay cells are actually excitatory^38^, but visual cortex also sends excitatory feedback to the thalamic reticular nucleus (TRN), a layer of inhibitory neurons adjacent to the thalamus, which can in turn inhibit LGN relay neurons. It thus seems plausible that direct corticothalamic excitatory feedback might influence LGN relay cells before the arrival of indirect inhibitory feedback from the TRN. Thus, even for connections between other areas than V1 and extrastriate visual cortex, predictive coding may present the same hypothesized temporal profile: excitation followed by inhibition.

Furthermore, even though inter-areal feedback connections are carried out predominantly via the excitatory neurons (since only they have long enough axons to connect different areas) and mostly target excitatory neurons^19^,^39^, the net effects of feedback are not always excitatory^12^. Hupé *et al*. showed that with very low saliency stimuli, cooling down V5, and thus interrupting its feedback, actually increased neural activities in V3^20^. Schneider *et al*. also revealed inhibitory effects of feedback in auditory cortex^40^. One possible mechanism for such inhibitory effects is excitatory cortico-cortical feedback reducing lower level activities by activating local inhibitory circuits^40^,^41^. This possible mechanism may help us reconcile our findings with neuroimaging results: one group of neurons in early visual cortex may be excited by the top-down prediction (i.e. the 3D shape); this enhancement could in turn activate the local inhibitory circuits to inhibit other groups of neurons, leading to an overall inhibitory effect. Since the excited neurons and the inhibited ones belong to different populations, this mechanism might result in a spatial dissociation of excitatory and inhibitory effects (rather than, or in addition to, the postulated temporal dissociation). Kok *et al*. provided evidence for such a spatial dissociation: they observed enhanced activity in the area where a Kanisza-like illusory shape was perceived, but reduced activity for the surrounding inducers^42^.

Predictive coding is a powerful scheme that describes perception as an inferential process “explaining away” predicted responses from input signals^3^,^4^. However, only limited experimental observations on this phenomenon are available. Based on these limited observations, several neuronal models of predictive coding have been put forward. Friston *et al*.^4^,^43^ built on Rao and Ballard’s original predictive coding model^3^ and proposed a specific distribution of functional roles across the cortical layers^44^. Spratling^6^ advocated a neuronal model with excitatory feedback which, according to our logic described before, fits better with the anatomical and neurophysiological evidence^17^,^18^,^19^,^20^,^21^.

As pointed out already by Spratling^6^, one possible way to dissolve the conceptual tension between classical models of feedback (e.g. biased competition) and predictive coding is by hypothesizing that all predictive coding schemes employ two types of neurons within each layer of the cortical hierarchy: prediction or representation units (P) and prediction error units (E). Feedback aims to inhibit the error units, but thereby also strengthens the representation at the lower level. Under some simplifying assumptions, this hypothesis makes classical models of biased competition and predictive coding mathematically equivalent^6^. In line with this notion, Kok *et al*. observed reduced overall activity for expected stimuli, yet an increased stimulus representation^45^. These findings are inconsistent with the idea that feedback globally inhibits sensory representations; rather, they support the notion that it is only the error units that are suppressed, and thereby predictions increase the signal-to-noise ratio.

In conclusion, the present psychophysical study suggests an excitatory influence of predictive feedback at the perceptual level. To build an optimal neuronal model of predictive coding, the consideration of the entire range of neuroimaging, neurophysiological and psychophysical evidence is necessary. We hope the observed excitatory influence of predictive feedback could thus help improve the design of future predictive coding models.

## Methods

### Subjects

Based on pilot experiments, we expected an average psychometric function shift of at least 5%, with a variability of 5% in the point of subjective equality of psychometric functions. To reach a statistical power of at least 95%, we determined that the sample size was twelve subjects. To monitor eye movements, we added two more subjects with an eye-tracker. Finally, fourteen volunteers (7 female, mean age 27.78 ± 3.78 years, one left handed, five with left eye dominance) participated in the main experiment.

Seven of these main experiment participants (4 female; mean age 28.5 ± 1.2 years; all right handed) performed the attention control experiment. Four main experiment participants and six other volunteers (10 participants, 5 female, mean age 28.1 ± 4.9 years) performed the “local features” control experiment. Seven main experiment participants (3 female, mean age 29.6 ± 4.2 years) performed the “response bias” control experiment. Two main experiment participants and three other volunteers (5 participants, 3 female, mean age 28.8 ± 2.2 years) performed the “same/different” control experiment. These sample sizes for control experiments were determined, based on the effect size obtained in the results of the main experiment, so as to ensure a minimum statistical power of 80% for each control experiment.

All subjects in the main experiment and all control experiments had normal or corrected to normal vision. The study was approved by the local ethics committee “Sud-Ouest et Outre-Mer I” and followed the Code of Ethics of the World Medical Association (Declaration of Helsinki). All subjects provided signed informed consent before starting the experiments.

### Apparatus

Stimuli were presented at 57 cm distance using a desktop computer (2.09 GHz Intel processor, Windows XP) with a cathode ray monitor (resolution: 800 × 600 pixels; refresh rate: 120 Hz, Gamma corrected luminance function). Stimuli were designed and presented via the Psychophysics Toolbox^46^ running in MATLAB (MathWorks).

### Stimuli and tasks

Twenty pairs of 3D-shape and random-lines stimuli were first generated. Similar to Murray *et al*. (2002), 3D-shapes were generated by randomly selecting 4–6 vertices, connecting the vertices and adding small extensions to render perceived depth. Random-lines stimuli were created by breaking the 3D-shape at its intersections and randomly shifting the lines (crossings were avoided) within the display. The diameter of both 3D-shape and random-lines stimuli was 3 degrees. In all experiments except the “local features” control experiment, the stimulus outlines were black. In the “local features” control experiment, these outlines were white.

Main experiment. Stimuli consisted of a central white fixation point (diameter: 0.2 degrees of visual angle) and two circular gray disks (diameter: 4 degrees each). One 3D-shape stimulus was in the center of one disk (3D-shape disk) and one random-lines in the other (random-lines disk). The disks were presented at an eccentricity of 3 degrees randomly on either side (left or right) of fixation. The luminance of the disks ranged from 20.17 cd/m^2^ to 32.3 cd/m^2^ (measured with a Minolta Chroma Meter CS-100, Minolta Co., Ltd, Osaka, Japan). To compute normalized luminance, the measured luminance values were divided by the middle value of the luminance range, i.e. 26.235 cd/m^2^. One disk had a fixed luminance level (100% normalized luminance) and the other a variable luminance value, randomly drawn from the normalized luminance set [80%, 84%, 88%, 92%, 94%, 96%, 98%, 100%, 102%, 104%, 106%, 108%, 112%, 116%, 120%]. Disks were presented on a black background (normalized luminance 0.0145%). Before stimulus onset, there was a blank screen that lasted from 200 to 800ms (random uniform distribution). The stimulus lasted for 750 ms and then the instruction “Which disk was brighter? Press the arrows” appeared on the screen until the subject’s response. Subjects were presented with 5 blocks of 200 trials, and asked to fixate the fixation point and use the arrow keys on a standard 105 key keyboard to respond (left arrow for left is brighter, right arrow for right is brighter). There was no feedback after the response. To monitor for breaks in fixation, eye movements of two subjects were recorded using a video-based eye tracker (EyeLink 1000 plus, SR Research, Ontario, Canada) with a sampling rate of 1000 Hz. The eye tracker was calibrated at the beginning of each block (only 4 blocks of 200 trials were performed by these subjects). For each trial, if the maximal deviation from fixation during stimulus presentation was bigger than 0.5 degrees from the fixation point, the trial was rejected automatically and the instruction “Please fixate on the fixation point” appeared on the screen.

Control experiment: attention. The fixation point was replaced by a rapid serial visual presentation (RSVP) stream of letters. The RSVP was made up of letters randomly drawn from the set [T, L, K, J, B, C, D], 2 degrees in diameter. Each letter was presented for 150 ms. A letter could not appear twice in a row, and the letter “T” appeared from one to four times (randomized from trial to trial). The RSVP sequence started before the disks presentation and ended after the disks. Fifteen letters were presented from time 0 to 2250 ms, while the disks were presented from time 750 to 1500 ms. The instruction “How many Ts were there? press the key of 1–4” appeared at time 2250 ms, until the subject’s response (using the keys 1,2,3,4 in the numeric keypad). There was a short beep sound feedback if subjects answered incorrectly in this task. After the subject’s response to the letter counting task, the instruction “Which disk was brighter? Press the arrows” appeared, and subjects performed the luminance judgment task as in the main experiment. Subjects were presented with 8 blocks of 100 stimuli. They were instructed that their primary task was to count the number of occurrences of the letter “T”.

Control experiment: local features. Stimuli were white 3D-shape and random-lines on a gray disk. The task was the same as in the main experiment.

Control experiment: response bias. Stimuli were the same as in the main experiment. The variable luminance value was randomly drawn from the normalized luminance set [80%, 86%, 92%, 96%, 98%, 100%, 102%, 104%, 108%, 114%, 120%]. The question given after each trial was: “Which disk was darker? Press the arrows”, and subjects were instructed to choose the darker disk using the arrow keys.

Control experiment: same/different judgment. Stimuli were the same as in the main experiment. The question given after each trial was: “Did the two disks have the same luminance?”, and subjects pressed one key to indicate that they perceived the same luminance and another key when they perceived a different luminance.

### Data analysis

The trials were classified into two categories: either the 3D-shape disk had a variable luminance value, or the random-lines disk had a variable luminance value. For all experiments except the same/different luminance experiment, for each trial type, the selection probability of the disk with the variable luminance value was computed, separately for each variable luminance value. Two psychometric functions were generated, one for each trial type, expressing the selection probability as a function of the variable luminance value, and fitted using normal cumulative distribution functions (each pair of psychometric functions was fitted with Gaussian cumulative functions with six parameters: mean and standard deviation separately for each psychometric function, and a common guess rate and lapse rate for both functions; the guess rate was set in the range of [0,1] and the lapse rate was set in the range of [0, 1-guess rate], which limited the maximum and minimum values of the psychometric functions to 1 and 0, respectively). Finally, we compared these two psychometric functions. The difference between the two psychometric functions at 50% selection probability was defined as the psychometric shift (and the difference between the two grand-average psychometric functions was defined as the average psychometric shift). A student’s t test against the null hypothesis of a psychometric shift equal to zero (both disks perceived equally bright) was performed using psychometric shifts from all subjects. For the same/different judgment, the probability of reporting “same luminance” was computed for each variable luminance value, and two psychometric functions were generated and fitted using Gaussian distribution functions, separately for each trial type. The difference between the peaks of the two Gaussian functions was defined as the psychometric shift in this experiment.

## Additional Information

**How to cite this article**: Han, B. and VanRullen, R. Shape perception enhances perceived contrast: evidence for excitatory predictive feedback? *Sci. Rep*. **6**, 22944; doi: 10.1038/srep22944 (2016).
